# Machine Learning-Guided Optimization of *p*-Coumaric Acid Production in Yeast

**DOI:** 10.1021/acssynbio.4c00035

**Published:** 2024-03-28

**Authors:** Sara Moreno-Paz, Rianne van der Hoek, Elif Eliana, Priscilla Zwartjens, Silvia Gosiewska, Vitor A. P. Martins dos Santos, Joep Schmitz, Maria Suarez-Diez

**Affiliations:** † Laboratory of Systems and Synthetic Biology, Wageningen University & Research, 6708 WE Wageningen, The Netherlands; ‡ Department of Science and Research, dsm-firmenich, Science & Research, 2600 MA Delft, The Netherlands; § Bioprocess Engineering Group, Wageningen University & Research, Wageningen 6700 AA, The Netherlands

**Keywords:** machine learning, DBTL, one-pot library, *Saccharomyces cerevisiae*

## Abstract

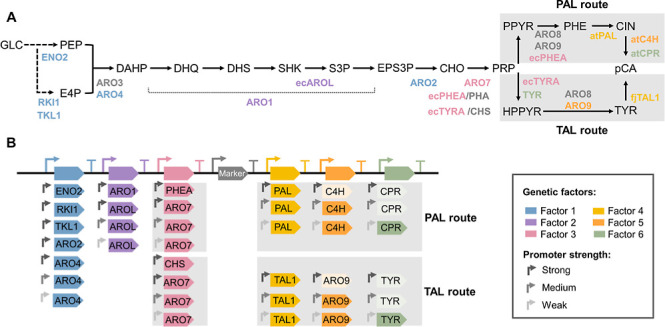

Industrial biotechnology uses Design–Build–Test–Learn (DBTL) cycles to accelerate the development of microbial cell factories, required for the transition to a biobased economy. To use them effectively, appropriate connections between the phases of the cycle are crucial. Using *p*-coumaric acid (pCA) production in *Saccharomyces cerevisiae* as a case study, we propose the use of one-pot library generation, random screening, targeted sequencing, and machine learning (ML) as links during DBTL cycles. We showed that the robustness and flexibility of the ML models strongly enable pathway optimization and propose feature importance and Shapley additive explanation values as a guide to expand the design space of original libraries. This approach allowed a 68% increased production of pCA within two DBTL cycles, leading to a 0.52 g/L titer and a 0.03 g/g yield on glucose.

## 1 Introduction

The reality of climate change calls for an imminent transition to a biobased economy less reliable on the petrochemical industry. Biotechnology contributes to solutions to this problem as metabolic engineering allows microbial production of a wide variety of compounds such as pharmaceuticals, biofuels, food additives, and bulk chemicals.^1^ However, these solutions often require very long development times that limit their real-world application.^2^

Design–Build–Test–Learn (DBTL) cycles offer a framework for systematic metabolic engineering. Pathways are designed during the Design phase, and strains are constructed in the Build phase and screened for production during the Test phase. In the Learning phase, a relationship between the pathway design and production is established and used to inform new DBTL cycles.^3^ Advances in synthetic biology and automation facilitate the engineering of microorganisms and increase the throughput of the Build and Test phases. However, predicting the effect of modifications in the Design phase that may lead to improvements is nontrivial.^4,5^ In fact, the acceleration of the Build and Test phases of the DBTL cycle might lead to a paradox where more data leads to more complexity but not necessarily better strain performance.^6^ To avoid this, an efficient and meaningful link between the Design and Learn phases of the cycle is crucial.

Machine learning (ML) can identify patterns in the system of interest without the need of a detailed mechanistic understanding of the problem.^7^ It has been used to aid strain development with applications ranging from gene annotation and pathway design to process scale-up.^4^ When used for pathway optimization, common approaches start by creating libraries of strains with varying regulatory elements (e.g., promoters and ribosome binding sites). These libraries include a defined solution space that can be explored by random or rational sampling.^8,9^ A subset of the library is then screened, and genotype and production data are used to train the ML algorithms. The algorithms then suggest a new round of (improved) strains for construction, effectively linking the Learn and Design phases of sequential DBTL cycles.^5,8,10−12^ Besides, ML algorithms are robust to missing data caused by unsuccessful construction of specific strains, which facilitates effective and efficient implementation of the DBTL cycles.^12,13^

**Figure 1:**
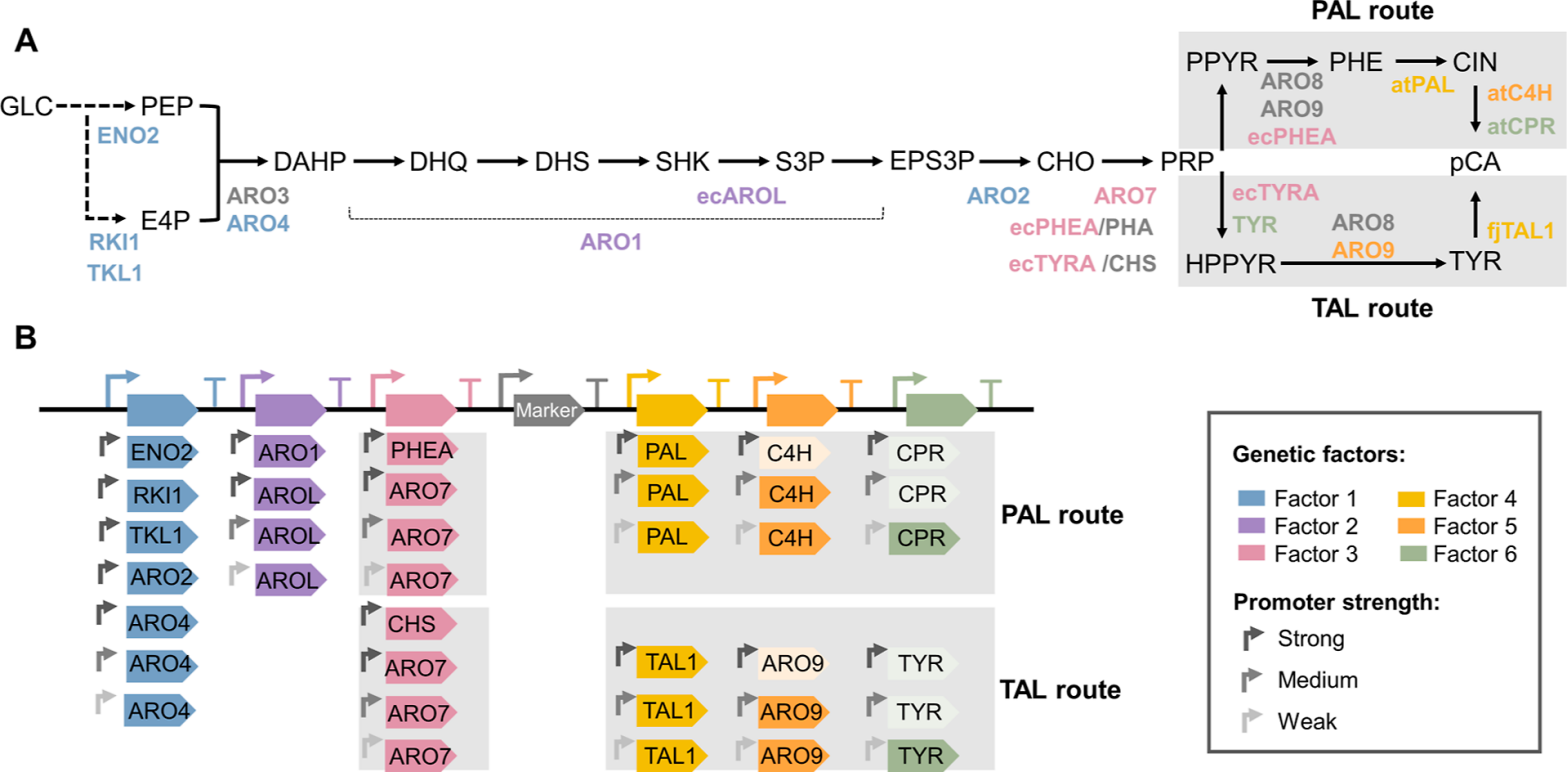
(A) pCA production pathway. Heterologous genes are preceded by a two-letter code indicating the organism of origin where “ec” refers to *E. coli*, “at” to *Arabidopsis thaliana*, and “fj” to *Flavobacterium johnsoniae*, see legend for color codes. (B) Library structure. The library consists of gene clusters formed by a selection marker (Marker) and six factors with levels including different open reading frames (ORFs) and different promoters. Lighter colors are used to indicate factor’s levels included in the design but not obtained experimentally. GLC, glucose; PEP, phosphoenolpyruvate; E4P, erithrose-4-phosphate; DAHP, 3-deoxy-7-phosphoheptulonate; DHQ, 3-dehydroquinate; DHS, 3-dehydroshikimate; SHK, shikimate; S3P, shikimate-3-phosphate; EPS3P, 5-enolpyruvylshikimate-3-phosphate; CHO, chorismate; PRP, prephenate; PPYR, phenylpyruvate; PHE, phenylalanine; CIN, cinnamate; pCA, *p*-coumaric acid; HPPYR, 4-hydroxyphenylpyruvate; TYR, tyrosine.

*p*-Coumaric acid (pCA) is an aromatic amino-acid-derived molecule produced from phenylalanine (Phe) or tyrosine (Tyr). It is naturally found in plants and serves as a starting material for commercially valuable products such as pharmaceuticals, flavors, fragrances, and cosmetics.^14^ In *Saccharomyces cerevisiae*, Phe and Tyr are synthetized via the prephenate pathway (Figure 1A).^15,16^ This pathway starts with the condensation of erythrose-4-phosphate (E4P) and phosphoenolpyruvate (PEP) by 3-deoxy-7-phosphoheptulonate synthase (ARO3/4). Then, the pentafunctional protein ARO1 converts 3-deoxy-7-phosphoheptulonate (DAHP) to 5-enolpyruvylshikimate-3-phosphate (EPS3P), which is converted to chorismate (CHO) by ARO2, and to prephenate (PRP) by ARO7. Prephenate can then be converted to Phe by prephenate dehydratase (PHEA) and ARO8/9 or to Tyr by prephenate dehydrogenase (TYR) and ARO8/9. To continue the synthesis of pCA, expression of heterologous genes is needed: Tyr ammonia lyase (TAL) for synthesis from Tyr or Phe ammonia lyase (PAL), cinnamate 4-hydroxylase (C4H), and its associated cytochrome P450 reductase (CPR) for synthesis from Phe.^14,17−19^

The prephenate pathway is highly regulated where Tyr exerts feedback inhibition on ARO3 and ARO7 and Phe on ARO4.^16^ This regulation together with the availability of precursors and appropriate expression of heterologous genes have been demonstrated to influence pCA production.^14,17,18^ However, testing the effect of these factors individually might result in the exclusion of possible synergistic effects. Alternatively, combinatorial optimization of metabolic pathways can facilitate the search for optimal production albeit involving the construction and testing of an exponentially growing number of strains.^8^

We used ML-guided DBTL cycles to improve pCA production in *S. cerevisiae*. We created combinatorial libraries based on the Tyr- or Phe-derived pathways that simultaneously altered expressed coding sequences and regulatory elements (promoters) (Figure 1B). We showed a better performance of the Phe-derived pathway, which was further optimized based on ML predictions. Following this strategy, we achieved a 68% improvement in production within two DBT(L) cycles and a final pCA titer of 0.52 g/L, resulting in a 0.03 g/g yield of pCA on glucose. Although higher pCA yields of up to 0.15 g/g have been previously obtained,^14^ this study is an example of the use of ML-guided DBTL cycles to systematize the generation of efficient strains.

## 2 Results

### 2.1 DBTL Cycle 1: Exploring the Design Space

#### 2.1.1 Design: Selection of Factors and Levels

Two independent libraries were designed depending on whether pCA was produced from Phe (PAL route) or Tyr (TAL route; Figure 1A). Any design of the libraries is formed by a 7-genes cluster (6 factors and a selection marker) integrated in the genome of *S. cerevisiae* (Figures 1B and S1). The combination of a promoter, open reading frame (ORF), and terminator (cassette) in the gene cluster constitutes a factor that can take different levels depending on the chosen promoter and/or ORF. The size of the library is determined by the number of factors and levels, so *library* = ∏_*i* = 1_^*F*^*L*_*i*_, where *F* is the number of factors and *L*_*i*_ is the number of levels of factor *i*. Both libraries shared factors 1 and 2 and differed in the other 4 factors (Figure 1B).

Factor 1 contained five ORFs: enolase (ENO1), ribose-5-phosphate isomerase (RKI1), transketolase (TKL1), ARO2, and feedback-resistant ARO4 (ARO4^K229L^) under the TDH3 promoter. Besides, ARO4^K229L^ could be downstream of two additional promoters (RPL8A and MYO4) as the expression of this gene has resulted in significantly increased pCA titers.^14,18^ ENO1, RKI1, and TKL1 were chosen considering that the availability of PEP and E4P can also affect the production. ARO2 was included as an additional level to test the effect of other shikimate pathway genes (Table 1).

**Table 1:** Summary of Factors and Their Levels in the TAL and PAL Libraries

factors	levels (promoter + ORF)						
	1	2	3	4	5	6	7
1	TDH3-ENO2	TDH3-RKI	TDH3-TKL	TDH3-ARO2	TDH3-ARO4	RPL8A-ARO4	MYO4-ARO4
2	TEF1-ARO1	TEF1-AROL	RPL28-AROL	UREA3-ARO4			
3	PRE3-PHA PRE3-CHS	PRE3-ARO7	ACT1-ARO7	PFY1-ARO7			
4	ENO2-PAL ENO2-TAL	RPS9A-PAL RPS9A-TAL	VMA6-PAL VMA6-TAL				
5	KI_OLE1-C4H KI_OLE1-ARO9	CHO1-C4H CHO1-ARO9	PXR1-C4H PXR1-ARO9				
6	PGK1-CPR PGK1-TYR	RPS3-CPR RPS3-TYR	CCW12-CPR CCW12-TYR				

Levels for factor 2 were based on the assumption of ARO1 as the rate-limiting step (Table 1). Rodriguez et al. observed increased pCA production when ARO1 or AROL from *Escherichia coli*, which catalyzes the phosphorylation of shikimate, was overexpressed in yeast.^18^ Therefore, 4 levels were chosen: expression of AROL under three different promoters (TEF1, RPL28, and UREA3) and expression of ARO1 under a strong promoter (TEF1).

The focus of factor 3 was on the expression of the feedback-resistant variant ARO7^G141S^ under three different promoters (PRE3, ACT1, and PFY1) as expression of this gene improved pCA titers.^14,18^ Besides, the expression of PHEA and TYRA from *E. coli* with the PRE3 promoter is considered as additional levels for the PAL and TAL libraries, respectively (Table 1). These bifunctional enzymes have a chorismate mutase activity (conversion of CHO to PRP) and either prephenate dehydratase (PHEA) or dehydrogenase (TYRA) activity, specific for the formation of Phe or Tyr, respectively.^20,21^

Factors 4, 5, and 6 of the PAL library each focused on one of the heterologous genes required for pCA production from Phe: PAL, C4H, and CPR under the control of three different promoters (ENO2, RPS9A, VMA6; KI_OLE1, CHOI, PXR1; and PGK1, RPS3, and CCW12, respectively). In the TAL library, levels of factor 4 were formed by TAL under the control of three promoters (ENO2, RPS9A, and VMA6). In order to obtain a design space with the same size as the PAL library, factors 5 and 6 included the expression of ARO9 and TYR with the same promoters used for the PAL library (Table 1).

Considering the factors and levels used, the number of possible designs in each library was 3024 (7·4·4·3·3·3).

#### 2.1.2 Build and Test: Construction and Screening of the Combinatorial Library

For each of the promoter–terminator pairs designed, the cassettes formed by the promoter-GFP-terminator were constructed and transformed into yeast. Positive colonies were found for all of the constructs but the strong promoter–terminator pairs for factors 3 and 5 (PRE3-ADH1 and KI_OLE1-TDH3). Cells were grown in BioLector bioreactors, and fluorescence was analyzed using fluorescence-activated cell sorting (FACS). For factor 1, the fluorescence of strong and medium promoters differed by an order of magnitude. For factors 2, 4, and 6, the fluorescence values for the medium promoters were approximately half of those from strong promoters. Weak promoters showed fluorescence values 1 or 2 orders of magnitudes below those of the strong and medium promoters (Figure S2).

The cassettes required for the in vivo assembly of the gene clusters were created by combining promoters, ORFs, terminators, and homology regions. All cassettes except those containing the strong promoter for factor 5 and the strong and medium promoters for factor 6 were obtained, which reduced the size of the PAL and TAL libraries from 3024 possible designs to 672 designs per library (Figure 1B).

**Figure 2:**
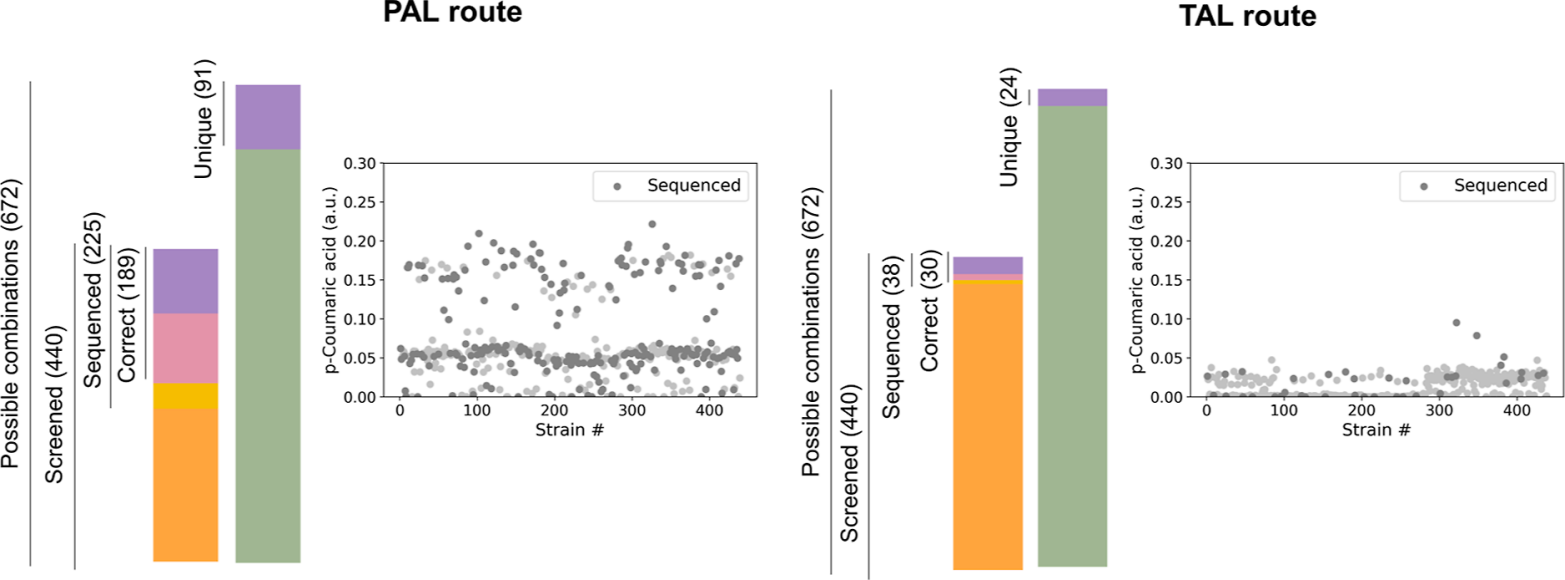
Screening before sequencing strategy. For each of the routes allowing pCA production, 672-member libraries were defined. For the PAL route, production of 440 randomly selected strains was measured, and 225 strains were selected for sequencing; 189 correct strains containing 91 unique pathway designs were found. For the TAL route, 440 strains were screened from which 38 were sequenced; 30 of these strains were correct, and 24 unique designs were found.

*S. cerevisiae* cells expressing Cas9 were transformed with a mixture of the correct cassettes using a one-pot transformation. Cells were plated in selective media, and 440 strains per library were randomly selected for screening of pCA production. These stains were grown in 96 DWP for 48 h, pCA was extracted, and samples were measured using nuclear magnetic resonance (NMR) (Figure 2). Colonies from the PAL route produced pCA ranging from 0 to 0.22 au; colonies from the TAL route produced significantly less pCA, with only three colonies producing above the detection limit (0.05 au) and a maximum production of 0.10 au.

**Figure 3:**
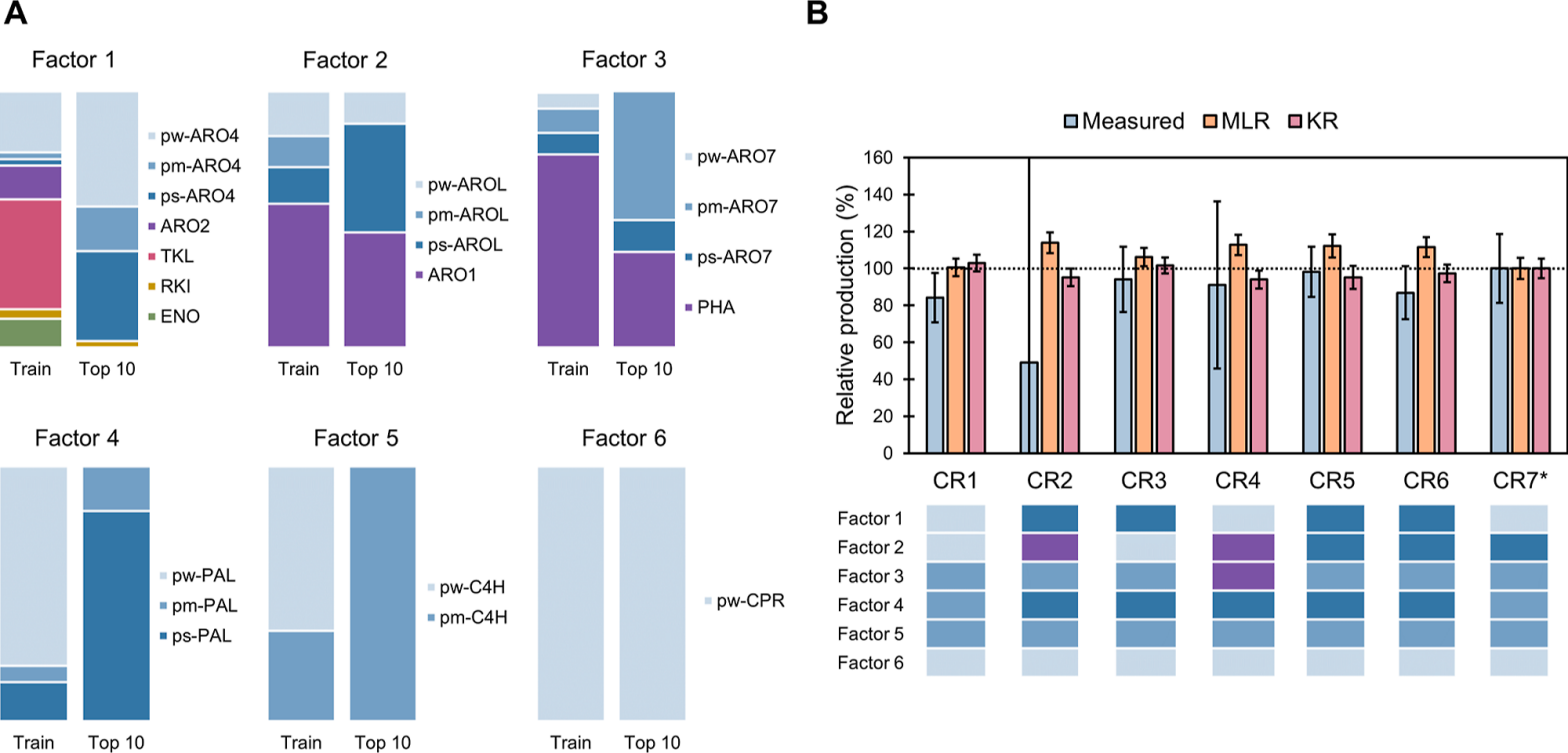
(A) Comparison of factor levels on the training set and top 10 predicted strains considering the CO (complete data set, one-time training), CR (complete data set, recurrent training), PO (producers data set, one-time training), and PR (producers data set, recurrent training) rankings. Ps, pm, and pw indicate strong, medium, and weak promoters, respectively. (B) Experimental validation of the CR ranking predictions. Production relative to the BMP strain (same as CR7* strain) is shown. The genotype of the strains follows the same color code presented in panel A.

Considering the screening results, the production space was sampled including low, medium, and high producers in order to obtain high-quality data for ML^4^ and to analyze the efficiency of the library generation method. For the TAL route, 38 strains were sequenced from which 30 sequences were correct (i.e., contained the gene cluster with 7 genes), and 24 contained unique pathway designs (i.e., different integrated gene clusters) (Figure 2). Considering that 80% of the sequenced strains were correct, the observed low pCA production was likely caused by the lower efficiency of the TAL route and not by incorrect library construction. These results agreed with previous reports that identified the PAL route as the most suitable pathway for pCA production.^14^ Therefore, optimization of pCA production was focused on the PAL route. Out of the 672 possible designs in this library, 225 strains were selected for sequencing based on their pCA titers, ensuring that the strains from different clusters were sequenced. We found 189 correct strains (84%) from which 91 (48%) were unique, validating the library construction approach (Figure 2). Out of the 91 unique designs, 58 designs were present in one strain, and 33 had multiple replicates (Figure S3). Besides, for all factors, at least a strain containing each of the levels was found (Figure 3A).

#### 2.1.3 Learn: Model Selection, Training, and Predictions

One of the challenges of applying ML to strain design is training data requirements. While some reports suggest the homogeneous sampling of the complete solution space,^4^ others suggest the benefit of including mainly good producers.^10^ Therefore, we divided our data into two data sets: the *complete* data set that included data from producers and nonproducers and the *producers* data set. Stratification was used during training to ensure a constant proportion of poor, medium, good, and very good producers in the training and test sets. Train sets were used to find optimal hyper-parameters for four ML algorithms: multiple linear regression (MLR), support vector regression (SVR), kernel ridge regression (KRR), and random forest regression (RFR). While MLR assumes a linear relationship between the factors and the response, SVR and KRR can capture nonlinear relationships, and random forest is an ensemble method that excels at handling complex interactions. The performance of the models with optimized hyper-parameters was evaluated on the test set (Figure S4). The models trained with the producers data set showed better performance than that of those trained using the complete data set (Table 2). MLR and KRR or all models were chosen as predictors for the complete and the producer data sets, respectively.

**Table 2:** Performance of ML Methods (*R*^2^) Trained with the Complete or Producer Datasets Using Stratification on Test Data^a^

	complete data set	producers data set
	(91 designs)	(63 designs)
MLR	0.70 ± 0.17	0.82 ± 0.15
SVR	0.71 ± 0.23	0.82 ± 0.19
KRR	0.72 ± 0.18	0.80 ± 0.11
RFR	0.72 ± 0.19	0.82 ± 0.16

a MLR, multiple linear regression; SVR, support vector regression; KRR, kernel ridge regression; RFR, random forest regression.

The selected models were trained in each data set using two different learning strategies: “*one-time training*” and “*recurrent training*” (Figure S4). The first strategy consisted of one-time training with all of the available data and did not provide uncertainty in the predictions. The second strategy was based on recurrent learning on 90% of the available data, which reduced the impact of possible outliers in the training data and allowed uncertainty quantification of predictions. The trained models were then used to predict the pCA titers of the 672 designs from the full design space. Considering that the models selected for each data set had similar performances (Table 2), the designs were ranked based on the frequency in which each design was predicted to be in the top 1, top 5, or top 10 by each model. In this way, the construction of designs commonly predicted as top producers by different models was favored. Four rankings were obtained: the CO and CR rankings based on the complete data set and the one-time or recurrent training strategies, respectively, and the PO and PR rankings based on the producers data set (Figure S4).

Training strategies were evaluated based on their ranking of the best-measured producer (BMP) strain, the five best-measured producers (5-BMPs), and all the measured nonproducers (Figure S5). The best measured producers were expected to rank high, while measured nonproducers were expected to hold lower positions. Regardless of the training strategy, including nonproducers during training did not change predictions of measured top producers but improved predictions of measured nonproducers, ensuring correct coverage of the complete design space by the ML predictions.

In order to improve pCA production, the designs predicted to render the highest titers were evaluated (Figure S6). Notably, the BMP strain was predicted as part of the top 10 designs in all but the CO ranking. A comparison between the levels present in the training data and the top 10 designs predicted by all of the learning strategies is depicted in Figures 3A and S6. Top predicted strains showed a preference for ARO4 under weak or strong promoters compared to the other ORFs. For factors 2 and 3, ARO1 or AROL under a strong promoter and PHA or ARO7 under its medium promoter were favored. Finally, the strongest promoters tested for PAL and C4H were enriched in the predicted top producers.

However, predicted pCA production improvements compared to the BMP strain were low (6 ± 8%, 2 ± 5%, 3 ± 6%, and 1 ± 5% depending on the learning strategy used) (Figure S7). Therefore, the initially screened library was a good representation of the whole design space and had already achieved the highest possible production. Although we used 13.5% of the full library data for model training, ML algorithms are frequently trained with data representing ca. 5% of the library design space. Therefore, we tested the effect of reducing data availability on model performance (Figure S12, Table 3). When 40% of the available training data was used for training with stratification (equivalent to 5.4% of the library space), the coefficients of determination of the test sets remained above 0.6 for all the models but MLR regardless of the data set used, suggesting that the identification of 36 unique strains could have been sufficient for ML training.

**Table 3:** Performance of ML Methods (*R*^2^) on Test Sets When Models Are Trained with Training Data Size Equal to 5.4% of the Library^a^

	complete data set		producer data set	
	no stratification	stratification	no stratification	stratification
MLR	0.52 ± 0.24	0.56 ± 0.23	0.61 ± 0.25	0.65 ± 0.23
SVR	0.57 ± 0.25	0.65 ± 0.21	0.64 ± 0.27	0.73 ± 0.22
KRR	0.61 ± 0.22	0.67 ± 0.16	0.65 ± 0.21	0.64 ± 0.19
RFR	0.65 ± 0.22	0.70 ± 0.20	0.70 ± 0.24	0.75 ± 0.22

a MLR, multiple linear regression; SVR, support vector regression; KRR, kernel ridge regression; RFR, random forest regression.

### 2.2 DBT Cycle 2: Expansion of the Original Design Space

ML analysis suggested that the optimal production is possible considering the initial design space had already been found. In order to validate this prediction, the top predicted designs by all of the learning strategies were constructed. Figure 3B shows the predicted and measured production of the top 7 designs in the CR ranking. As expected, the production of these strains did not significantly improve, with respect to the BMP strain. Similar results were obtained with the top strains from the CO, PO, and PR rankings, with production remaining within the BMP mean ±20% (Figure S8).

**Figure 4:**
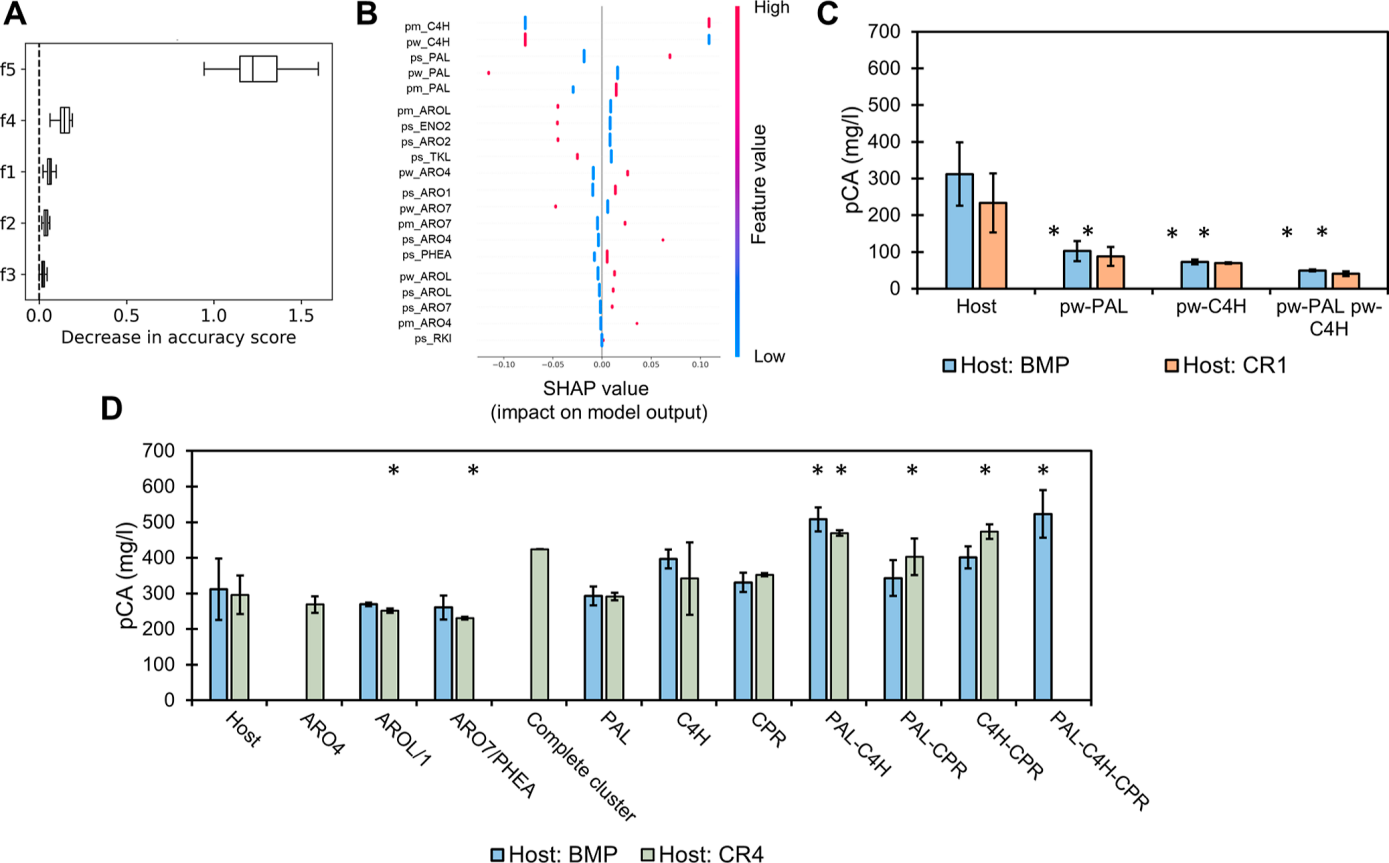
(A) Representative example of feature selection results, where f1, f2, f3, f4, and f5 refer to factors 1–5, respectively. (B) Representative example of the SHAP values, where ps, pm, and pw refer to strong, medium, and weak promoters, respectively. (C) Effect of substituting promoters of PAL and/or C4H by the weakest alternative (pw) in two different hosts: BMP and predicted top producer by the *complete recurrent* strategy (CR1). (D) Effect of integration of double copies of genes in two different hosts: BMP and the constructed predicted top producer by the *complete recurrent* strategy (CR4). Significant differences with respect to each host are indicated by *. Colonies with double copies of ARO4 and the complete gene cluster were not obtained in the BMP host. Colonies with double copies of PAL-C4H-CPR were not obtained in the CR4 host, and a single colony was obtained with the correct integration of the complete gene cluster.

In order to improve pCA production, the original design space had to be expanded, and the permutation feature importance and Shapley additive explanation (SHAP) values were used to guide the new designs. Permutation feature importance identifies the factors with the greatest influence on model performance by evaluating the decrease in model accuracy when the values of a factor are shuffled. Factor 5, representing the expression strength of C4H, was identified as the most relevant factor, followed by factor 4 (PAL expression) (Figures 4A and S10). Considering that the predicted top producers had C4H under the strongest promoter tested and never chose the weaker promoter for PAL, we hypothesized that higher expression of these genes could lead to higher production. This was confirmed by the SHAP values, a technique for explainable ML based on game theory that not only identifies significant factors but also determines how they affect the model output.^22^ For all the training strategies used (except the MLR model with the producer data set), the highest positive impact on model output (i.e., production) was caused by expressing C4H and PAL under the strongest promoter tested. Similarly, the highest negative impact was caused by the expression of C4H and PAL under weaker promoters (Figures 4B and S11). The importance of these genes was confirmed by substituting the promoters of PAL or C4H with weak promoters in the BMP strain and the best strain in the CR ranking (CR1). In both cases, strains with the lower expression of PAL and/or C4H showed significantly reduced pCA production (Figure 4C). Besides, although the effect of different expression levels of CPR could not be assessed due to unsuccessful cassette construction, changing the promoter of CPR in the BMP and CR1 strains did not significantly change pCA production (Figure S9).

To further increase the expression of the genes, the strains with double copies of each of the genes were created using BMP and the best-constructed strain from CR ranking (CR4) as hosts. Positive colonies containing double copies of ARO4 and ARO4-AROL-ARO7-PAL-C4H-CPR in the BMP host and PAL-C4H-CPR in the CR4 host were not found. As expected, when extra copies of factors 1 (ARO4), 2 (AROL or ARO1), and 3 (ARO7 or PHEA) were integrated, production of pCA did not significantly change (Figure 4D). However, production did not significantly increase when double copies of PAL or C4H were integrated. Even though average production increased with a double copy of C4H, this change was not significant (Figure 4D). The integration of a double copy of the complete gene cluster was achieved only in one colony of the CR4 host, and its production was similar to that in strains with an extra copy of C4H. In both hosts, double copies of PAL and C4H resulted in significantly increased production (63% in BMP and 58% in CR4). Besides, significantly increased production was also found when double copies of PAL-CPR (36%) and C4H-CPR (60%) were expressed in CR4 and PAL-C4H-CPR were expressed in BMP (68%) (Figure 4D).

The observed increase in pCA production, only obtained when expanding the original design space, confirmed that the original space had been sufficiently sampled and validated feature importance and SHAP values as strategies to guide its expansion.

## 3 Discussion

Accelerating the design of industrially relevant strains is crucial to transition to a biobased economy. In order to exploit the full potential of microorganisms, the combinatorial optimization of metabolic pathways is required. However, this involves the construction and testing of an exponentially growing number of strains which becomes unfeasible.^23^ Alternatively, the solution space can be sampled by following a rational or randomized approach. Statistical design of experiments reduces the number of strains to build and test while maximizing the information gained about the complete solution space. However, it requires the construction of specific strains, and it is sensitive to experimental limitations: information is lost when a strain cannot be built.^24^ As shown here, ML presents an alternative to learn from randomly generated libraries of strains, which is robust to missing data. Besides, when ML is used, libraries can be flexibly designed to include factors with different numbers of levels based on prior knowledge. We used factor 1 to explore genes that could influence pCA production assigning 7 levels to this factor. Instead, we assigned 3 levels to factors 4, 5, and 6, aiming to fine-tune the expression of the required heterologous genes. We used 4 levels for factors 2 and 3 to simultaneously test the effect of homologous ORFs from different origins and tune the expression of one of them. The robustness and flexibility of the ML approach were also shown when some of the designed levels could not be implemented experimentally. Although the design spaces of the PAL and TAL libraries were reduced from 3024 members to 672, the relationship between the remaining levels could still be efficiently explored.

Another challenge to combinatorial pathway optimization is the need for the characterization of genetic parts that ensure that the solution space is sufficiently explored. This is especially important when the aim is to fine-tune the expression levels of pathway genes.^5,8,10,12^ In principle, the optimization of gene expression would benefit from the use of quantitative variables as factors (e.g., GFP fluorescence, protein levels) as they would allow the identification of an optimal expression level.^25^ However, although effort is taken to appropriately characterize how regulatory elements affect gene expression, this is seldom achieved as in vivo expression depends on factors such as the downstream gene^26^ or the gene order in an operon^3^ and cannot be accurately predicted. Alternatively, regulatory elements can be treated as categorical variables reducing the impact of the characterization data.^8^ This approach allowed us to include noncharacterized promoters as members of the library and avoid a further decrease in the design space size. Besides, the use of categorical variables does not limit factor levels to differences in expression strength. As shown here, factors might include levels that represent differences in expression but also different ORFs, broadening the scope of ML-guided pathway optimization to the selection of genes from different origins or alternative overexpression targets.

A limitation to the use of ML is the requirement for sufficient and quality data for training.^4^ We showed that including nonproducers as part of the training set is not required to find the top producing strains but improves predictions of poor producers which helps ensuring that the design space has been sufficiently sampled. This is especially important when the top producer is already present in the training data. Although we trained ML models with data representing 13.5% of the library, we showed that when the amount of data used for training decreased, stratification during training improved the mean *R*^2^ and reduced its standard deviation (Figure S12, Table 3). Stratification allowed the classification of samples based on production. Therefore, a sufficient number of samples from each category should be present in the training data. As shown here, this can be achieved using a screening before sequencing approach, which allowed an efficient exploration of the design space and reduced the chance of sequencing duplicate designs.

ML algorithms cannot extrapolate; they cannot predict the performance of strains with factor levels different from those used during training.^7^ Still, they can be used to determine whether the best producer from the library is already present in the training data and to justify the expansion of the original design space. When this is required, we proposed the use of feature importance and SHAP values to guide this expansion and point at the most relevant factors, which, in this case, led to a 68% improvement in pCA production. Notably, while feature importance only points as the significant factors, the SHAP values provide additional information regarding how the factor’s levels influence the model output.^22^

The highest titers of pCA measured in this study were 0.51 ± 0.03 and 0.52 ± 0.06 g/L obtained using the BMP strain with additional copies of PAL-C4H or PAL-C4H-CPR. These strains were cultivated in 96DWP with 20 g/L of glucose, resulting in 0.03 g/g pCA yield on glucose. However, higher titers and yields of pCA have been reported. Rodriguez et al. obtained 1.96 g/L of pCA (0.04 g/g) by expressing AROL, feedback-resistant variants of ARO4 and ARO7, eliminating competing metabolic pathways and using synthetic fed-batch media.^18^ Production was further improved by Liu et al. by combining the TAL and PAL pathways and including a phosphoketolase pathway to increase the E4P availability. This strain produced 3.1 g/L in shake flasks and up to 12.5 g/L in bioreactors operated as fed-batch with a maximum yield of 0.15 g/g.^14^ Considering these results, the production of our developed strains could be improved in next cycles that focus on gene deletions and media and bioprocess optimization. This optimization would benefit from an improved experimental throughput achievable, for instance, using barcode sequences to mitigate sequencing costs^27^ or a pCA biosensor for titer estimation.^28^ This throughput, in turn, could allow the simultaneous testing of gene deletions, process conditions, and gene overexpression using multiple gene copies that could lead to a further increased production. However, a tradeoff between the build and test capacity and efficiency and the complexity of the learning step must be established by ensuring that a minimum percentage of the library space (e.g., 5%) can be used for model training. When this throughput is not achievable, sequential DBTL cycles, such as those presented here, are useful to identify the relevance of the tested factors and levels and decide whether they are maintained or replaced in subsequent optimization cycles.

This study is an example of how ML-guided DBTL cycles can accelerate the generation of efficient strains. We showed the robustness of this approach to experimental limitations and its flexibility regarding design, which can be expanded beyond traditional tuning of gene expression. We propose a screening before sequencing approach to allow for stratification during training, especially important for small data sets. Furthermore, we showed how feature importance and SHAP values can be used to expand the original design space and further improve strain performance.

## 4 Materials and Methods

### 4.1 Organisms and Media

*S. cerevisiae* strains were derived from CEN.PK113-7D and grown at 30 °C in Yeast Extract Phytone Dextrose media for transformations and precultures (YEPhD, 2% Difco phytone peptone (Becton-Dickinson (BD), Franklin Lakes, NJ, USA), 1% Bacto Yeast extract (BD), and 2% d-glucose (Sigma-Aldrich, St Louis, MO, USA)) and minimal media for production experiments (Verduyn Luttik with 2% glucose^29^). When required, antibiotics were added to the media at appropriate concentrations: 200 μg/mL nourseothricin (Jena Bioscience, Germany)and 200 μg/mL geneticin (G418, Sigma-Aldrich). *E. coli* DH10B (New England BioLabs, Ipswich, MA, USA) was used as the cloning strain and grown at 37 °C in 2*Peptone Yeast Extract media [2*PY, 1.6% tryptone peptone (BD), 1% Bacto yeast extract (BD) and 0.5% NaCl (Sigma-Aldrich)]. When required, antibiotics were added to the media at appropriate concentrations: 100 μg/mL ampicillin (Sigma-Aldrich) and 50 μg/mL neomycin (Sigma-Aldrich). Solid medium was prepared by the addition of Difco granulated agar (BD) to the medium to a final concentration of 2% (w/v).

### 4.2 Cassette Construction

DNA templates for promoters and terminators^30^ as well as ORFs were codon optimized^31^ and can be found in Table S1. Bricks were assembled into cassettes (promoter + ORF + terminator) via Golden Gate (using BsaI-HF v2.0 (NEB) and T4 DNA Ligase (Invitrogen)) into a backbone plasmid containing a 50 bp homologous connector sequence to facilitate in vivo recombination of the gene cluster as described in ref (32) (Figure S1). Golden Gate products were transformed into chemically competent *E. coli* DH10B. The Wizard SV 96 Plasmid DNA purification system (Promega, Madison, WI, USA) was used for plasmid isolation. The cassettes were confirmed by polymerase chain reaction (PCR) using the Q5 High-Fidelity DNA polymerase (NEB) with primers from IDT (Leuven, Belgium) and analyzed on a LabChip GX Touch Nucleic Acid Analyzer (Perkin-Elmer). Plasmids with correct fragment size were amplified by PCR using Q5 High-Fidelity DNA polymerase (NEB), and integration site flanks (50 base pair homologous region) were attached to the first and the last cassettes of the gene cluster (Figure S1). PCR products were purified using Promega Wizard SV PCR Clean-Up kit and quantified using DropSense 96 (Trinean).

### 4.3 Strain Construction

Strains were constructed as described in refs (32,33). In short, host strain SHK001 preexpressing Cas9 (integrated on locus INT1, Table S1) was used to enable a targeted integration via CRISPR-Cas9 into *S. cerevisiae*’s genome. A linear guide RNA targeting a single locus (Table S5) was amplified from a gBlock (IDT) with 50 bp homology regions to pRN1120. Plasmid backbone pRN1120 was amplified for in vivo assembly of the gRNA plasmid. PCRs were performed with Q5 High-Fidelity DNA polymerase (NEB). PCR products were confirmed on a 0.8% agarose gel and purified using Wizard SV Gel and PCR Clean-Up kit (Promega). DNA fragments were quantified using a Nanodrop (Thermo Fisher Scientific). The primers used are provided in Table S4.

Equimolar amounts (100–300 ng/kb) of the cassettes, linear gRNA (210 ng/kb), and linear backbone (35 ng/kb) fragments were transformed to the cells following the LiAc/ssDNA/PEG (lithium acetate/single-stranded DNA/polyethylene glycol) method.^34^ Reagents required for yeast transformation were obtained from Sigma-Aldrich (lithium acetate dihydrate (LiAc) and DNA sodium salt from salmon testes (ssDNA)) and Merck (poly(ethylene glycol) 4000 (PEG)). The connector sequences on the cassettes facilitate in vivo recombination of a cluster of genes in the genome.^32^ Transformants were plated on a Qtray (NUNC) containing YEPhD agar medium and a selection agent. The colonies appeared on the plate after 3 days of incubation at 30 °C. Single colonies were picked with Qpix 420 (Molecular Devices) into 96-well plates containing YEPhD agar medium and a selection agent and regrown for 3 days at 30 °C.

### 4.4 Whole Genome Sequencing

*S. cerevisiae* cells (OD 5–10) were pelleted and lysed in 200 μL of 0.9% physiologic salt supplied with 2 μL of RNase cocktail (Invitrogen) and 5 mg/mL Zymolyase 100T (MP Biomedicals). The mixture was incubated at 37 °C for 45 min. Next, 200 μL of 2X cell lysis solution (0.05 M EDTA, 4%SDS) was added to the mixture, followed by vortexing. 168 μL of protein precipitation solution (10 M NH4Ac) was added, and proteins were precipitated by centrifugation for 10 min at 20 K rcf at 4 °C. The DNA in the supernatant was precipitated with an equal volume of isopropanol (centrifugation for 2 min at 16 K rcf at room temperature). The DNA pellet was washed with 70% ethanol. The ethanol was discarded, and the pellet was left to dry and then dissolved in MilliQ water. The isolated genomic DNA was quantified using the Qubit (Thermo Fisher Scientific) and Nanodrop (Thermo Fisher Scientific), purified using the Zymo Research gDNA Clean & Concentrator kit and sequenced using the ligation sequencing kit (LSK-SQK109) with the native barcoding expansion (EXP-NBD114) from Oxford Nanopore Technologies according to the manufacturer’s instructions on a GridION device (FLOW-MIN106 flow cell).

### 4.5 Promoter–Terminator Characterization

Combinations of promoter–terminators were characterized using GFP as a reporter gene (see Table S3 for details). Precultures were prepared in 96-well half-deep well plates (HDWPs) containing 350 μL of YEPhD + Pen/Strep (Invitrogen) and incubated at 30 °C, 750 rpm, 80% humidity for 48 h. Ten μL of the grown preculture was reinoculated to an MTP-R48-B FlowerPlate (m2p-laboratories) containing 1 mL of minimal medium + Pen/Strep (Invitrogen). The plate was incubated 48 h in the BioLector at 30 °C, 800 rpm, and 85% humidity. Biomass (em. 620 nm/ex. 620 nm) and fluorescence (em.488 nm/ex. 520 nm), each with 3 filters (gain of 100, 50, and 20), were measured every 15 min. 40 μL of 2 day old main culture was measured using fluorescence-activated cell sorting (BD, FACSAria Fusion) to detect the single cells expressing GFP at a flow rate of 10 000 evt/s. The signal of fluorescent proteins was detected with a bandpass filter set at 530/30 nm for eGFP. The data was recorded using the BD FACSDiva 8.0.2 software to retrieve the geometric mean of the fluorescence distribution. Data was analyzed using FlowJo (version 10.6.2).

### 4.6 pCA Production Experiments

Colonies were grown in 96 microtiter plates (MTP) Nunc flat bottom (Thermo Fisher Scientific) containing YEPhD and an appropriate selection agent for 48 h at 30 °C, 750 rpm, and 80% humidity. Cultures were reinoculated in HDWP (Thermo Fisher Scientific, AB-1277) containing 350 μL of YEPhD and a selection agent and grown for 48 h in the same conditions. The grown cultures were reinoculated to HDWP containing 350 μL minimal media (Verduyn Luttik with 2% glucose^29^) and incubated for 2 days at 30 °C, 750 rpm, 80% humidity. In all plates, blank wells and wells containing a control strain (SHK0046, see Table S3) were included. For flow-NMR measurements, 250 μL of broth was sampled to a 96-deep well plate (DWP) and mixed with 500 μL of acetonitrile (Sigma-Aldrich) by pipetting. The mixture was centrifuged at 4000 rpm for 10 min. 500 μL of the supernatant was transferred to a new DWP for analysis with flow-NMR. For liquid chromatography–mass spectrometry (LC/MS) measurements, 250 μL of broth was sampled. One ml acetonitrile was added, and the sample was mixed by pipetting and centrifuged. 250 μL of the supernatant was diluted with 375 μL of MilliQ and used for analysis with LC/MS.

### 4.7 pCA Quantification with Automated Segmented-Flow NMR Analysis

The DWP plates were lyophilized to remove the nondeuterated solvents. 100 μL of the solution of 1 g/L internal standard, 1,1-difluoro-1-trimethylsilanyl methylphosphoric acid (FSP, Bridge Organics), in MilliQ water was added into DWP prior to the lyophilization. To the lyophilized samples was added 600 μL of D_2_O (Cambridge Isotope Laboratories (DLM-4)) and homogenized. The samples were analyzed on a CTC PAL3 Dual-Head Robot RTC/RSI 160 cm robotic autosampler (CTC Analytics AG, Zwingen, Switzerland) fluidically coupled to a Bruker spectrometer Avance III HD 500 MHz UltraShield.^35^^1^H spectra were recorded with the standard pulse program (zgcppr) with the following parameters: 16 scans, 2 dummy scans, 33 000 data points, 16.4 ppm spectral width, 1.2 s relaxation delay (d1), 8 μs 90° pulse, 2 s acquisition time, 15 Hz water suppression, and a fixed receiver gain (rg) of 64 Spectra were processed and analyzed using Topspin 4.1.4 (Bruker). Spectral phasing was applied, and spectra were aligned to 3-(trimethylsilyl)-1-propanesulfonic acid-d6 sodium salt (DSS-d6, Sigma-Aldrich) at 0 ppm. Auto baseline correction was applied on the full spectrum width. Additional third-order polynomial baseline correction for selected regions was applied if needed. The amount of pCA (doublet, 6.38 ppm, n = 2H) was calculated relative to the signal of FSP. NMR production data per plate was normalized by SHK0046 production.

### 4.8 pCA Quantification with LC-HR-MS Spectrometry

Samples were analyzed on a Vanquish Horizon UHPLC system coupled to a Q Exactive Focus mass spectrometer (Thermo Fisher). The chromatographic separation was achieved on an Acquity UPLC BEH C18 column (100 mm × 2.1 mm, 1.7 μm, Waters), using gradient elution with (A) 0.025% formic acid in LC–MS-grade water and (B) 90% LC–MS-grade acetonitrile (Sigma-Aldrich) with 10% mobile phase A with a run time of 9 min. The gradient started with 1% B linearly increased to 50% B in 5 min, followed by rapid increase to 99% B in 0.1 min, kept at 99% for 1.9 min, and then re-equilibrated with 1% B for 1.9 min. The flow rate was kept at 0.6 mL/min using an injection volume of 2 μL, and the column temperature was set to 50 °C. pCA was detected in negative APCI mode and quantified using an external calibration line of a reference standard. Using this chromatographic system, the coumaric acid elutes at retention times of 3.05 min with *m*/*z* 163.0403 (M-H), in good agreement (within 2 ppm) with the theoretical *m*/*z* value of 163.04007.

### 4.9 ML-Guided Strain Design

Originally, the PAL and TAL libraries each contained 3024 different designs. However, due to problems during cassette construction, the design space was reduced to 672 designs per library. We randomly screened 440 strains per library and classified them into four clusters based on NMR pCA production titers. Strains from every cluster were randomly selected for sequencing in order to cover the complete solution space. Colonies were considered correct when they had targeted integration of the complete gene cluster (7 cassettes, one per factor, and the selection marker). For gene clusters present in more than one correct sequenced colony, the average pCA production was considered. Two data sets were used: a *complete data set* including data from producers and nonproducers and a *producers data set*. Colonies were considered nonproducers when the measured pCA production was below 0.05 au.

From the available regressor models in the scikit-learn library, the performance of MLRs, SVRs, RFR, and KRR models was evaluated. pCA production was modeled using the factor levels (genes or their expression strength), treated as categorical variables using one-hot encoding, as inputs (Table 1). Models were evaluated on their ability to predict pCA titers measured by NMR (model output). Each data set was split into train (90% data) and test sets (10%) using stratification (i.e., maintaining the proportion of the different classes in both sets). For all models except MLR, hyper-parameters were selected based on leave-one-out cross-validation in the train set using the maximum error as the score. Predictions of models with optimized hyper-parameters were compared to the test set using the coefficient of determination (*R*^2^) as the score. This process was repeated ten times, and models were compared based on their average *R*^2^ on the test sets. Additionally, the impact of the training data size on model performance was tested: after the training test split, percentages of the training data from 5 to 100% were used for training, and model performance was evaluated using the test set with *R*^2^ as the score. See Figure S4 for an overview of the model selection strategy.

For each data set, the models selected based on *R*^2^ were trained following two different strategies: “*one time training*” and “*recurrent training*”. In the first strategy, all data from the data set was used for model training. In the second strategy, 90% of the data from the data set was used for model training, and this process was repeated 100 times. Trained models were used to predict pCA titers for all of the designs in the design space. For each data set and training strategy, top producers were ranked based on the frequency of each design being predicted as top 1, top 5, and top 10 by each model (Figure S4).

The impact of the different factors on pCA production was evaluated by permutation feature importance using the permutation_importance function from the inspection module of the scikit-learn library. In addition, the SHAP values were calculated using the shap library.^22^

All of the data and scripts used are available in GitLab (https://gitlab.com/wurssb/Modelling/ml4pca). Model selection, training, and feature importance were performed using Python 3.8.8 and Scikit-learn 1.1.3.^36^

## Supporting Information Available

Scripts used during this study for the analysis of the screening and sequencing data, model selection, and hyperparameter tuning; evaluation of training data size on model performance, model training and predictions; and analysis of feature importance and SHAP values are available in Gitlab (https://gitlab.com/wurssb/Modelling/ml4pca). List of promoters, ORFs and terminator sequences; list of integration sites used for strain construction; lists of strains, primers, and plasmids used in this study; features of sgRNA and crRNA design; overview of library design and construction; results of the promoter–terminator characterization with GFP; pCA production of the correct strains from the PAL library; schematic representation of model selection and training strategies; ranking of BMP, top 5 μ_B_P, and worst producers; genotype of the top 10 predicted strains by each learning strategy; prediction of pCA production of the top 10 predicted strains by each learning strategy; experimental validation of ML predictions; pCA production of strains with additional CPR promoters; feature importance and SHAP analysis results; and effect of training data size on model accuracy (PDF)
Quantitative information of the promoter–terminator characterization using GFP; validation of the learning from the first DBTL including top predicted producers and negative controls; and pCA production of strains constructed in the second DBT cycle (expansion of design space) (XLSX)
List of promoters, ORFs, and terminator sequences used in the study: integration sites used for strains construction; list of strains used in this study and their genotype; list of plasmids used in this study; list of primers used in this study; and sgRNA design (XLSX)
