# Supplementary material for: Machine Learning-Guided Optimization of p-Coumaric Acid Production in Yeast
Source: ACS Synth Biol. 2024 Mar 28;13(4):1312–22. doi: 10.1021/acssynbio.4c00035 (PMC11036487; doi:10.1021/acssynbio.4c00035)
Supplement: Supplementary file 1 — sb4c00035_si_001.pdf [file sb4c00035_si_001.pdf]

Supporting Information

Machine learning-guided optimization of p-coumaric acid production in yeast

Sara Moreno-Paz*[1], Rianne van der Hoek*[2], Elif Eliana[1], Priscilla Zwartjens[2], Silvia Gosiewska[2], Vitor A.P. Martins dos Santos[3], Joep Schmitz#[2], Maria Suarez-Diez#[1]

*These authors contributed equally. #These authors are the corresponding authors. Emails: joep.schmitz@dsm-firmenich.com; maria.suarezdiez@wur.nl
[1]Laboratory of Systems and Synthetic Biology, Wageningen University \& Research, 6708 WE Wageningen, The Netherlands.
[2]Department of Science and Research - dsm-firmenich, Science \& Research, 2600 MA Delft, The Netherlands.
[3]Bioprocess Engineering Group, Wageningen University \& Research, Wageningen 6700 AA, The Netherlands.

TABLE OF CONTENTS

**Supplementary Tables**	
Table S1: Promoters, ORF and terminator sequences	2
Table S2: Integration sites used for strains construction	2
Table S3: List of strains used in this study	2
Table S4: List of plasmids used in this study	2
Table S5: List of primers used in this study	2
Table S6: Features of sgRNA and crRNA design	3
**Supplementary Figures**	
Figure S1. Library design and construction	4
Figure S2. Promoter-terminator characterization	4
Figure S3. pCA production correct strain PAL library	5
Figure S4. Model selection and training strategies	5
Figure S5. Ranking of BMP, top 5 BMP and worst producers	6
Figure S6. Genotype top 10 predicted strains all learning strategies	7
Figure S7. Phenotype top 10 predicted strains all learning strategies	8
Figure S8. Validation ML predictions	9
Figure S9. Additional promoters CPR	10
Figure S10. Feature importance	10
Figure S11. SHAP values	11
Figure S12. Effect of training data size on model accuracy	12
**Supplementary References**	12

Table S1: Promoters, ORFs and terminator sequences

See attached Excel file.

Table S2: Integration sites used for strains construction.

Integration site	Chromosome	Location	Experiment
INT1	15	Non-coding region between NTR1 (YOR071c) and GYP1 (YOR070c)	Cas9 integration
INT69B		(Genbank: AEHG01000256.1)	DBTL Cycle 1
INT72B		(Genbank: AEHG01000557.1)	DBTL Cycle 2

Table S3: List of strains used in this study.

See attached Excel file.

Table S4: List of plasmids used in this study.

Plasmid	Genotype	Reference	Addgene
pCSN061	*CEN6/ARS TRP1* KanMX bla Kl11p-SpCas9-GND2t	(2)	# 101725
pRN1120	2µm NatMX amp^R	(2)	# 101750

Table S5: List of primers used in this study.

Name	Sequence	Purpose
pSH001	CTTATCGATACCGTCGACCTCGAGGGGGGGCCCGGTACCCAGCTTTTGTTCCGCGGTCTTTGAAAAGATAATG	Amplification of Cas9 sgRNA expression cassette (RV)
pSH002	AAAATACAACAAATAAAAAACACTCAATGACCTGACCATTTGATGGAGTTCCGCGGAGACATAAAAAAC	Amplification of Cas9 sgRNA expression cassette (FW)
pSH003	AACTCCATCAAATGGTCAGG	Amplification of Cas9 sgRNA recipient plasmid pRN1120 (FW)
pSH004	AACAAAAGCTGGGTACCGGG	Amplification of Cas9 sgRNA recipient plasmid pRN1120 (RV)
		Amplification of INT69B left flank (RV) with homology to conX
		Amplification of INT69B right flank (FW) with homology to conX
Add primers used to amplify cassettes		

Table S6: sgRNA design.

Target Locus	GBlock sequence
INT69	TACCCAGCTTTTGTTCCGCGGTCTTTGAAAAGATAATGTATGATTATGCTTTCACTCATATTTATACA<br>GAAACTTGATGTTTTCTTTCGAGTATATACAAGGTGATTACATGTACGTTTGAAGTACAACTCTAGAT<br>TTTGTAGTGCCCTCTTGGGCTAGCGGTAAAGGTGCGCATTTTTTCACACCCTACAATGTTCTGTTCAA<br>AAGATTTTGGTCAAACGCTGTAGAAGTGAAAGTTGGTGCGCATGTTTCGGCGTTCGAAACTTCTCCGC<br>AGTGAAAGATAAATGATCAAACAACAGTTCCGTAACCGGTTTTAGAGCTAGAAATAGCAAGTTAAAAT<br>AAGGCTAGTCCGTTATCAACTTGAAAAAGTGGCACCGAGTCGGTGGTGCTTTTTTTGTTTTTTATGTC<br>TCCGCGGAACTCCATCAAATGG
INT72	TACCCAGCTTTTGTTCCGCGGTCTTTGAAAAGATAATGTATGATTATGCTTTCACTCATATTTATACA<br>GAAACTTGATGTTTTCTTTCGAGTATATACAAGGTGATTACATGTACGTTTGAAGTACAACTCTAGAT<br>TTTGTAGTGCCCTCTTGGGCTAGCGGTAAAGGTGCGCATTTTTTCACACCCTACAATGTTCTGTTCAA<br>AAGATTTTGGTCAAACGCTGTAGAAGTGAAAGTTGGTGCGCATGTTTCGGCGTTCGAAACTTCTCCGC<br>AGTGAAAGATAAATGATCGATGCTGACACAATTAATGTGTTTTAGAGCTAGAAATAGCAAGTTAAAAT<br>AAGGCTAGTCCGTTATCAACTTGAAAAAGTGGCACCGAGTCGGTGGTGCTTTTTTTGTTTTTTATGTC<br>TCCGCGGAACTCCATCAAATGG

Primer homology

SNR52p and ATG

Target sequence

Sup4T

A

Factors	Possible promoters	Levels (promoter + ORF)							Terminator	# Cassettes (Prom + ORF + Ter)
		1	2	3	4	5	6	7		
1	ps = TDH3<br>pm = RPL8A<br>pw = MYO4	ps-ENO2	ps-RKI	ps-TKL	ps-ARO2	ps-ARO4	pm-ARO4	pw-ARO4	TEF2	7
2	ps = TEF1<br>pm = RPL28<br>pw = UREA3	ps-ARO1	ps-AROL	pm-AROL	pw-AROL				PGK1	4
3	ps = PRE3<br>pm = ACT1<br>pw = PFY1	ps-PHA<br>or<br>ps-CHS	ps-ARO7	pm-ARO7	pw-ARO7				ADH1	4
4	ps = ENO2<br>pm = RPS9A<br>pw = VMA6	ps-PAL<br>or<br>ps-TAL	pm-PAL<br>or<br>pm-TAL	pw-PAL<br>or<br>pw-TAL					TDH1	3
5	ps = Kl_OLE1<br>pm = CHO1<br>pw = PXR1	ps-C4H<br>or<br>ps-ARO9	pm-C4H<br>or<br>pm-ARO9	pw-C4H<br>or<br>pw-ARO9					TDH3	3
6	ps = PGK1<br>pm = RPS3<br>pw = CCW12	ps-CPR<br>or<br>ps-TYR	pm-CPR<br>or<br>pm-TYR	Pw-CPR<br>or<br>pw-TYR					GPM1	3

B

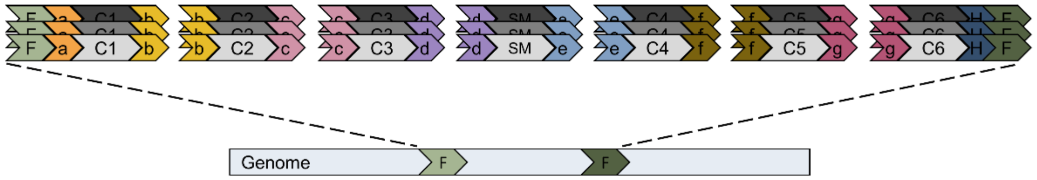

Figure S1: **A**. Cassettes used for library transformation. A cassette is a combination of promoter, ORF and terminator. Cassettes containing promoter-ORF combinations shown in orange could not be obtained. **B**. Schematic representation of the integration of a gene cluster. Connector sequences a to g represent homology regions for *in vivo* recombination of cassettes (C1 to C6) and the selection marker cassette (SM); flank sequences homologous to the genome integration site are shown as F.

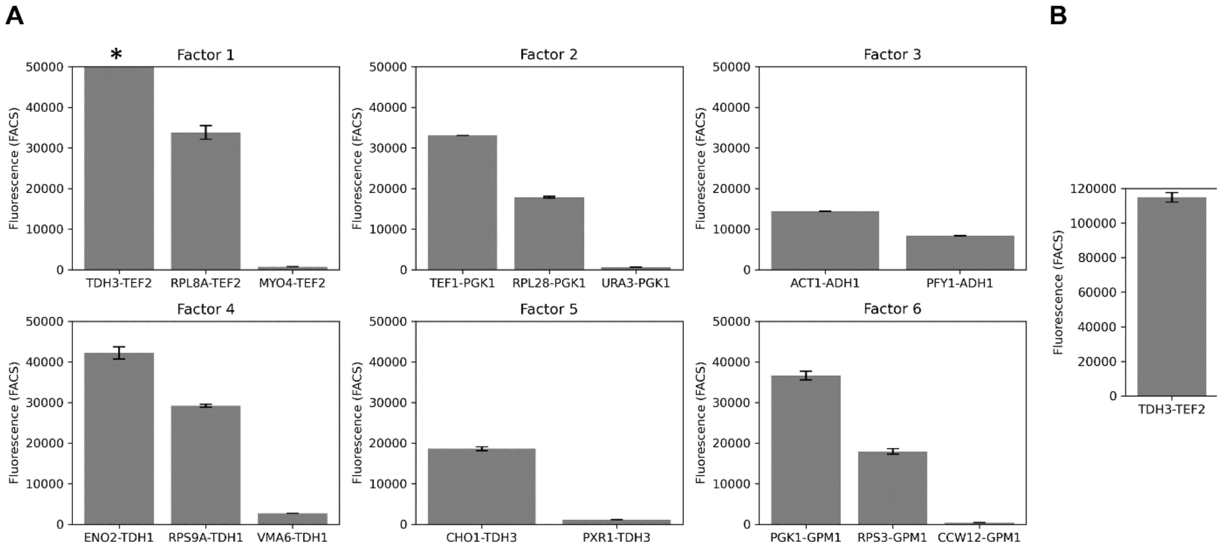

Figure S2: Promoter-terminator characterization by GFP fluorescence measured using fluorescence activated cell sorting (FACS) (**A**). Zoom-out for the fluorescence values (**B**).

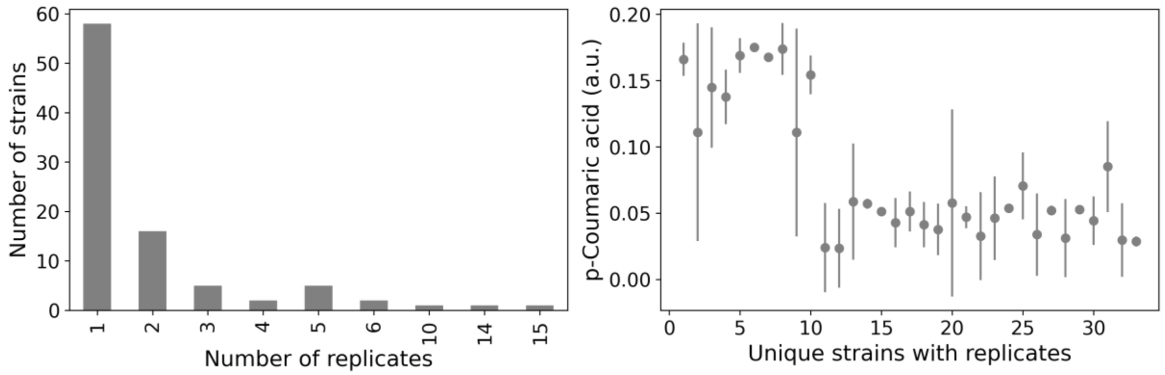

Figure S3: Characterization of the correct sequences from the PAL library. Frequency of strains with the same designs (left) and average $_{\rho}$CA production of designs with replicates (right).

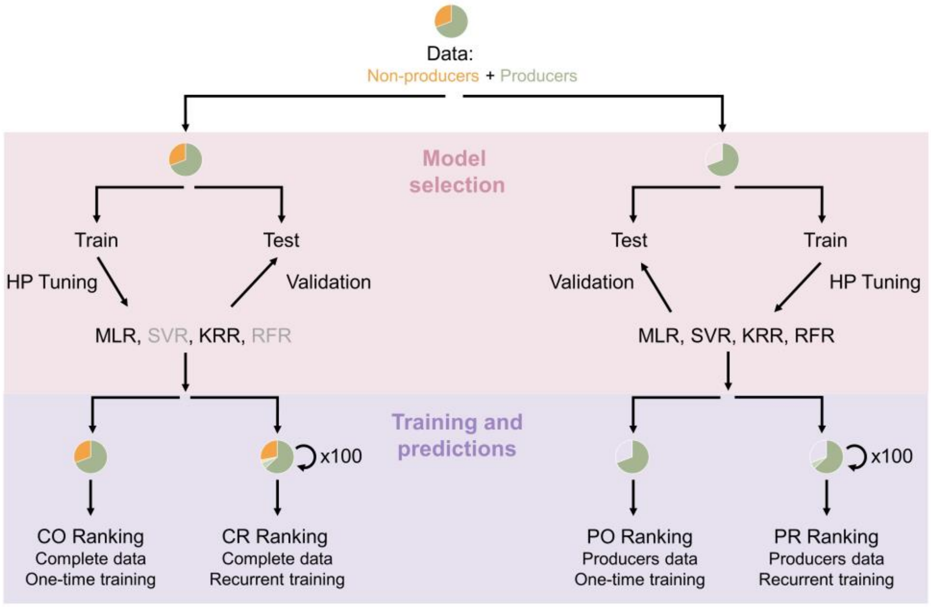

Figure S4: Model selection and training strategies. Genotype and production data were divided in two datasets: the *complete* and *producer* datasets that differ in the inclusion of data from nonproducers. Each dataset was used for hyper-parameter (HP) tuning of four ML models: multiple linear regressor (MLR), support vector regressor (SVR), kernel ridge regressor (KRR) and random forest regressor (RFR). Accuracy of models with optimal HP was evaluated on the test sets and is shown in the table. For each dataset, two learning strategies were applied: *one train training*, where all the data was used for training and *recurrent training*, where 90% of the training data was iteratively used for training.

	Best measured producer	Best 5 measured producers	Non-producers
**CO Ranking**	Predicted to be in:<br>- Top 12% (MLR)<br>- Top 0.9% (KRR)	Predicted to be in:<br>- Top 19% (MLR)<br>- Top 20% (KRR)	Predicted to be in:<br>- Bottom 46% (MLR)<br>- Bottom 40% (KRR)
**CR Ranking**	Predicted to be in:<br>- Top 8% (MLR)<br>- Top 2% (KRR)	Predicted to be in:<br>- Top 18% (MLR)<br>- Top 19% (KRR)	Predicted to be in:<br>- Bottom 44% (MLR)<br>- Bottom 40% (KRR)
**PO Ranking**	Predicted to be in:<br>- Top 5% (MLR)<br>- Top 15% (SVR)<br>- Top 4% (KRR)<br>- Top 0.1% (RFR)	Predicted to be in:<br>- Top 23% (MLR)<br>- Top 21% (SVR)<br>- Top 22% (KRR)<br>- Top 15% (RFR)	Predicted to be in:<br>- Bottom 52% (MLR)<br>- Bottom 60% (SVR)<br>- Bottom 59% (KRR)<br>- Bottom 60% (RFR)
**PR Ranking**	Predicted to be in:<br>- Top 6% (MLR)<br>- Top 10% (SVR)<br>- Top 4% (KRR)<br>- Top 3% (RFR)	Predicted to be in:<br>- Top 23% (MLR)<br>- Top 20% (SVR)<br>- Top 23% (KRR)<br>- Top 15% (RFR)	Predicted to be in:<br>- Bottom 50% (MLR)<br>- Bottom 52% (SVR)<br>- Bottom 58% (KRR)<br>- Bottom 61% (RFR)

Figure S5: Ranking of top measured producer, top 5 measured producers and non-producers based on four different training strategies. Results are given per model used in each strategy: MLR, multiple linear regressor; KRR, kernel ridge regressor; SVR, support vector regressor; RFR, random forest regressor. *Ranking of non-produces excludes design 560 which is predicted to produce by all models independently of the training strategy. The BMP was ranked in the top 0.1% to 15% depending on the model used and regardless of the training strategy. Similarly, the 5-BMPs were always predicted to be, at least, in the top 22% of the library. Measured non-producers were ranked in the bottom 46% or 60% of the library depending on the dataset used for training (*complete* or *producers* respectively). Therefore, including non-producers during training did not change predictions of top producers, but improved predictions of non-producers, ensuring correct coverage of the complete library by the ML predictions.

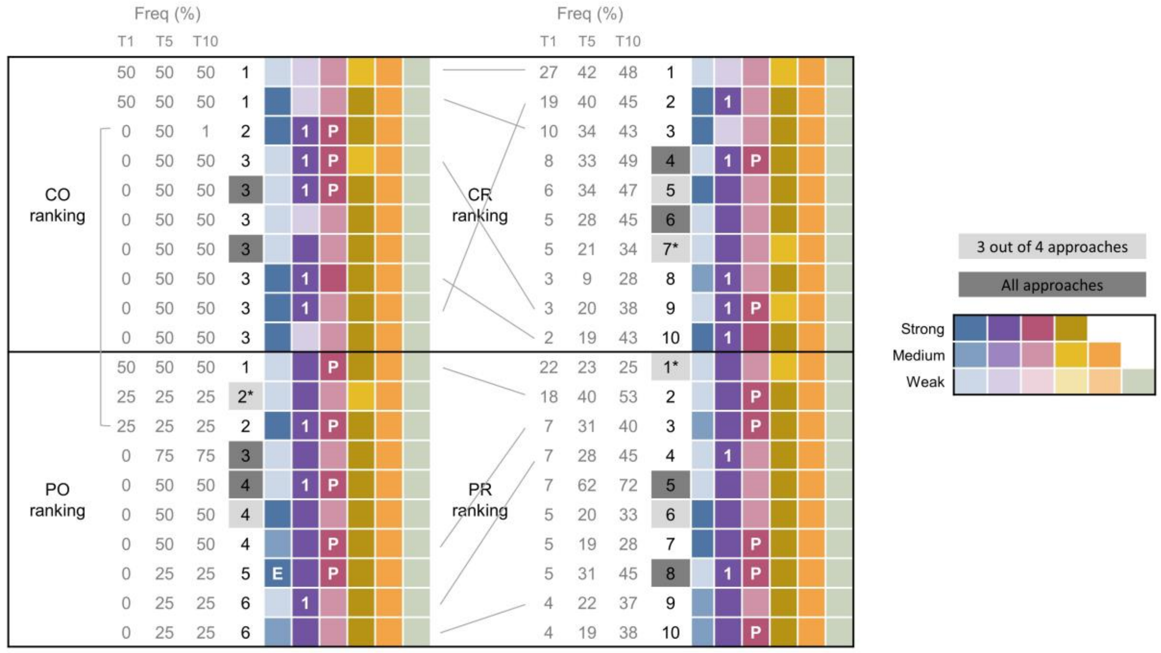

Figure S6: Summary of top 10 predicted producers by each learning strategy. Designs are ranked based on the frequency (Freq.) they are chosen as top 1 (T1), top 5 (T5) or top 10 (T10) by different models or data points included during training. Factor 1 refers to ARO4 except an E is shown (ENO1), factor 2 refers to AROL except a 1 is shown (ARO1), factor 3 refers to ARO7 unless a P is shown (PHEA), factor 4 refers to PAL, factor 5 refers to C4H and factor 6 refers to CPR. Predicted designs shared by different learning strategies are linked by lines or highlighted in grey. * indicates designs equal to the best measured producer strain (BMP strain).

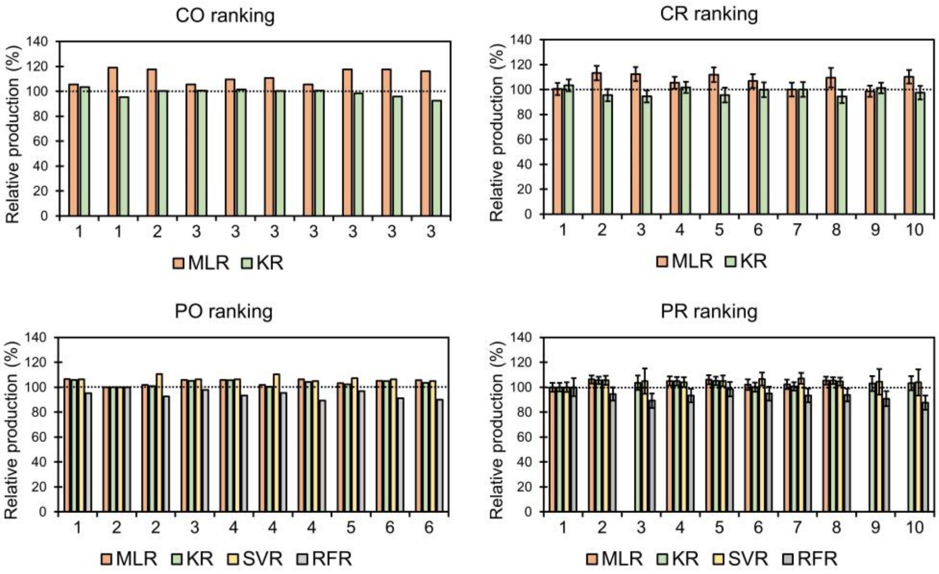

Figure S7: Predicted pCA production by the top 10 ranked strains found using four different leaning strategies. Production relative to the predicted production of the top measured producer is shown. CO, complete dataset with one time training; CR, complete dataset with recurrent training; PR, producers dataset with one time training; PR producers dataset with recurrent training.

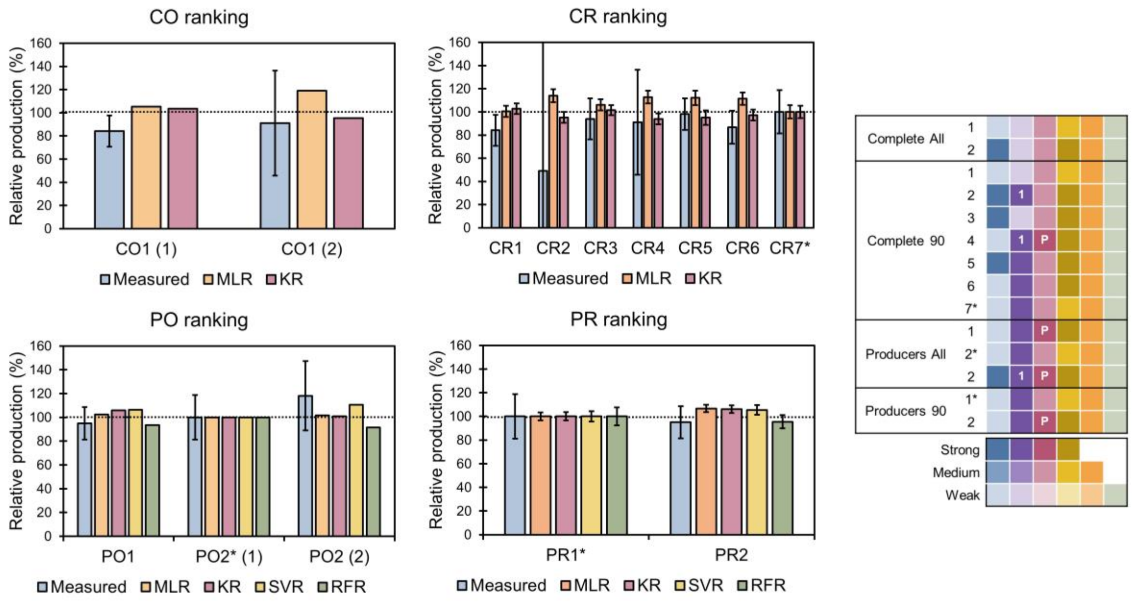

Figure S8: Validation of ML predictions. Comparison of measured and predicted relative production of the predicted top producers relative to the production of the best measured produced (BMP). Each of the left panels represents a different learning strategy . Genotypes of the plotted strains are shown in the right panel where * indicates strains equal to BMP. Factor 1 refers to ARO4, factor 2 refers to AROL expect when 1 is shown (ARO1), factor 3 refers to ARO7 except when P is shown (PHEA), factor 4 refers to PAL; 5 to C4H and 6 to CPR. Promoter strengths are represented by colour intensity. Strain names are defined based on the ranking they belong and their position in the ranking, when two strains share the same position they are followed by (1) and (2). CO, complete dataset with one time training; CR, complete dataset with recurrent training; PR, producers dataset with one time training; PR producers dataset with recurrent training.

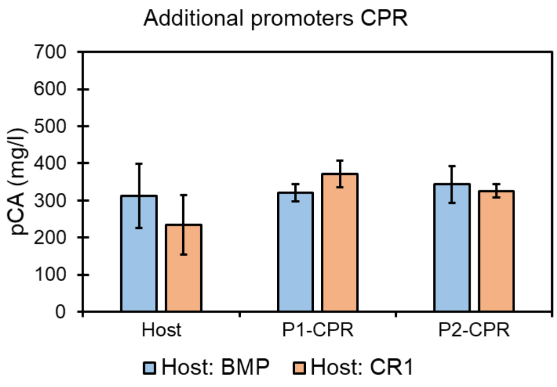

Figure S9: Effect of substituting the CPR promoter in two different hosts: the best measured strain (BMP) and the top producer in the CR ranking (complete dataset recurrent training).

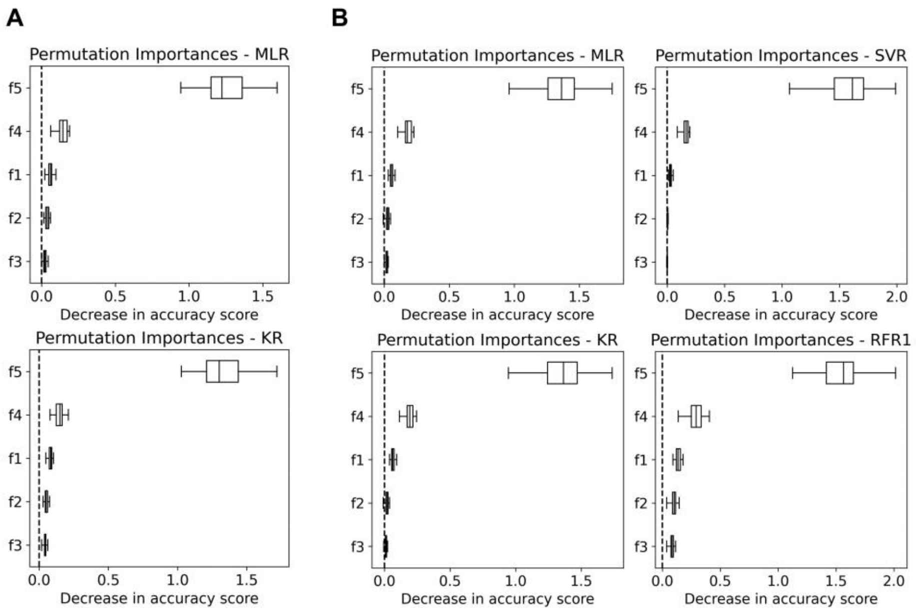

Figure S10: Permutation feature importance results obtained using the *complete* (**A**) or the *producers* (**B**) datasets.

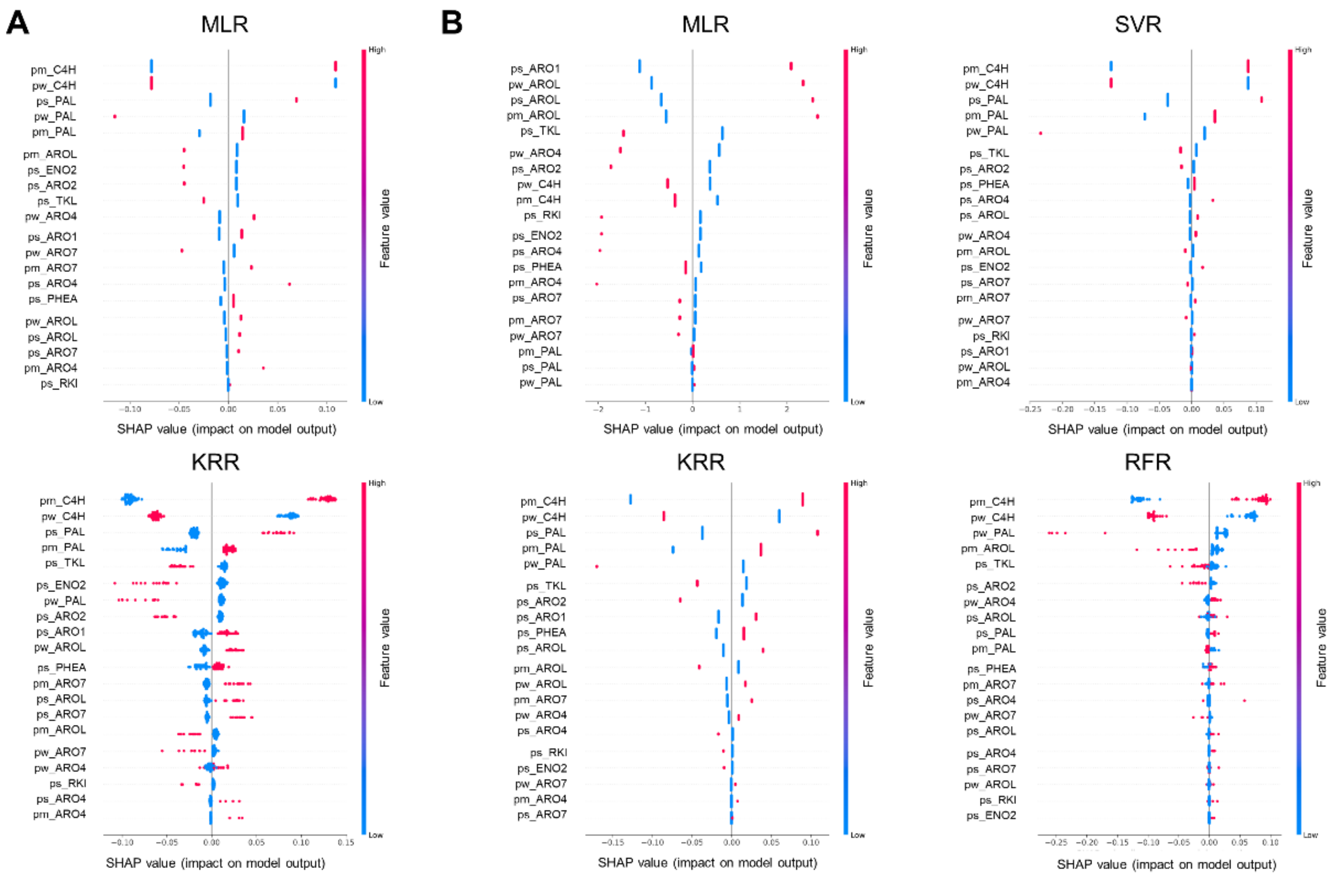

Figure S11: SHAP values obtained using the *complete* (**A**) or the *producers* (**B**) datasets.

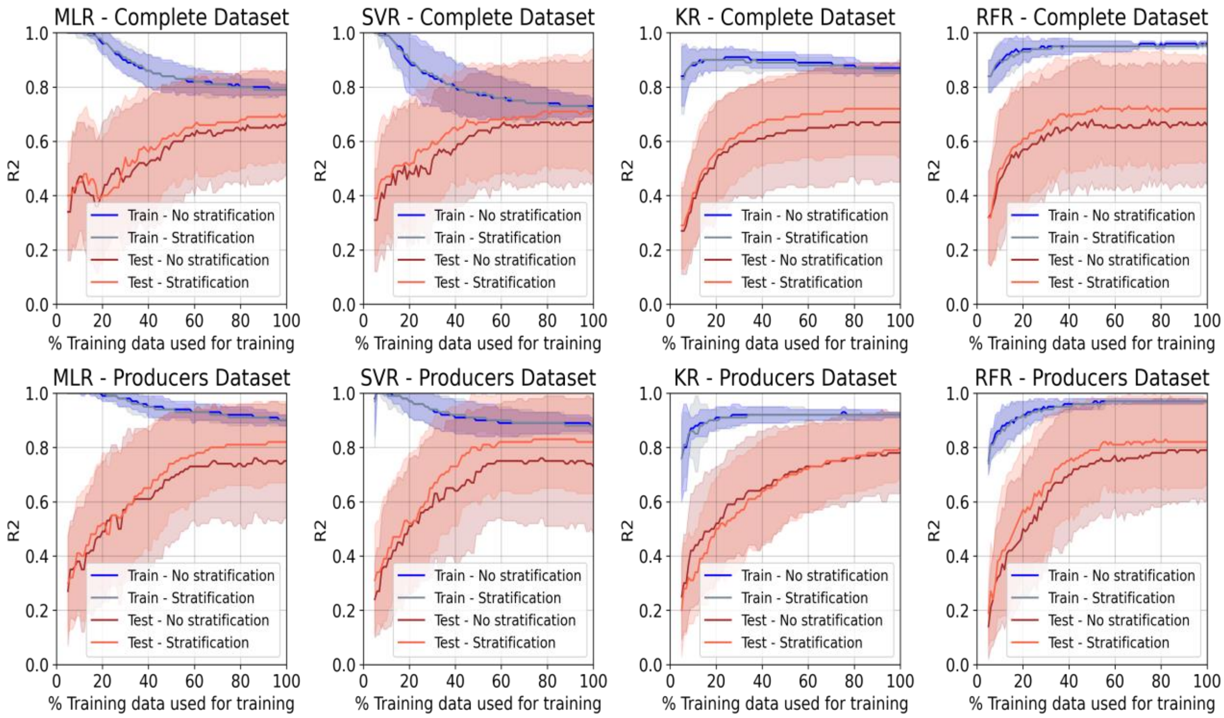

Figure S12: Effect of training data size on accuracy of predictions of different ML algorithms (MLR, multiple linear regression; SVR, support vector regression; KR kernel ridge regression; RFR, random forest regression) with the complete or producers dataset. Negative R^2 values obtained for some test-train splits were omitted for the calculation of mean and std (this values are obtained when the average of the training data is a better estimator than the trained model).

SUPPLEMENTARY REFERENCES

1. Van Dijken,J.P., Bauer,J., Brambilla,L., Duboc,P., Francois,J.M., Gancedo,C., Giuseppin,M.L.F., Heijnen,J.J., Hoare,M., Lange,H.C., *et al.* (2000) An interlaboratory comparison of physiological and genetic properties of four Saccharomyces cerevisiae strains. *Enzyme and Microbial Technology,* 26, 706-714.

2. Verwaal,R., Buiting-Wiessenhaan,N., Dalhuijsen,S. and Roubos,J.A. (2018) CRISPR/Cpf1 enables fast and simple genome editing of Saccharomyces cerevisiae. *Yeast*, 35, 201-211.
